# Prognostic utility of tumor-infiltrating lymphocytes in residual tumor after neoadjuvant chemotherapy with trastuzumab for HER2-positive breast cancer

**DOI:** 10.1038/s41598-018-38272-1

**Published:** 2019-02-07

**Authors:** Sasagu Kurozumi, Kenichi Inoue, Hiroshi Matsumoto, Takaaki Fujii, Jun Horiguchi, Tetsunari Oyama, Masafumi Kurosumi, Ken Shirabe

**Affiliations:** 1 Division of Breast Surgery, Saitama Cancer Center, Saitama, Japan; 2 Division of Breast Oncology, Saitama Cancer Center, Saitama, Japan; 3 Department of General Surgical Science, Gunma University Graduate School of Medicine, Gunma, Japan; 4 Department of Breast Surgery, International University of Health and Welfare, Chiba, Japan; 5 Department of Diagnostic Pathology, Gunma University Graduate School of Medicine, Gunma, Japan; 6 Department of Pathology, Saitama Cancer Center, Saitama, Japan

## Abstract

Predictive utility of tumor-infiltrating lymphocytes (TILs) in HER2-positive breast cancer patients receiving neoadjuvant chemotherapy (NAC) with concurrent trastuzumab remains unclear. We examined TILs grades of pretreatment cancer tissue specimens and residual tumors after NAC with trastuzumab and determined the predictive utility of the TILs grade in pathological complete response (pCR) and the prognostic power of TILs in HER2-positive breast cancer. This cohort study included 128 HER2-positive breast cancer who received NAC with trastuzumab. TILs grading of the tumor stroma in pretreatment biopsy specimens and residual tumors after NAC with trastuzumab was categorized as low, intermediate, and high based on the criteria of the International Working Group. In current study, the pCR rate was 64.8%, and the Relapse-free survival (RFS) was significantly worse in the non-pCR group than in the pCR group. The pCR rate correlated with the TILs grade in pretreatment tumors. In 45 non-pCR patients, TILs grade was higher in the residual tumors than in the pretreatment tumors. The RFS was significantly better in residual tumors with high TILs grade than those with low TILs grade (*p = *0.033). In conclusion, assessment of the TILs grade in residual tumors after NAC with trastuzumab might be necessary to determine patients with good prognosis among those who do not achieve pCR.

## Introduction

A variety of clinical trials on neoadjuvant chemotherapy (NAC) for breast cancer demonstrated its utility in both early-stage and advanced breast cancer. Evidence to date suggest that NAC in patients for whom mastectomy is unavoidable can significantly increase the number of patients eligible for breast-conserving surgery. Furthermore, various clinical trials representative of the National Surgical Adjuvant Breast and Bowel Project (NSABP) protocols B-18^1^ and B-27^2^ demonstrated that pathological complete response (pCR) was an exceptionally useful prognostic factor. As a result, recent clinical trials on new drugs for breast cancer also evaluated their efficacy by assessing preoperative drug therapy with pCR as an endpoint^3^. Approximately 20% to 30% of all breast cancers are HER2-positive, and NAC with trastuzumab, often administered in routine practice in HER2-positive breast cancer, achieves a high rate of pCR. We previously reported response rates of 65% or higher with NAC using either paclitaxel or docetaxel, which was followed by fluorouracil, epirubicin, and cyclophosphamide (FEC), concomitantly administrated with trastuzumab^4^.

Variations in immune responses within the cancer tissue are predicted to depend on the characteristics of immune cells that comprise tumor-infiltrating lymphocytes (TILs); these variations are also proposed to impact drug sensitivity and prognosis in breast cancer. Many retrospective studies suggested the potential utility of TILs expression in breast cancer as a prognostic factor and a predictor of the efficacy of drug therapy^5–10^. Among the clinical trials on adjuvant chemotherapy for breast cancer, the results of the Breast International Group (BIG) 2–98 trial^11^ suggested an association between TILs expression and the efficacy of anthracyclines in HER2-positive breast cancer. However, the drug effect of TILs in preoperative chemotherapy combined with trastuzumab and its utility as a prognostic factor in HER2-positive breast cancer remain controversial.

We therefore evaluated TILs expression in breast cancer tissue specimens obtained from patients before and after treatment with NAC plus trastuzumab. We also assessed the association of TILs expression with pCR and prognosis following combination treatment with NAC and trastuzumab.

## Results

### Patient and tumor characteristics

Patient and tumor characteristics are shown in Table S1. Median age of 128 patients was 53 years, and 77 (60.2%) patients were postmenopausal. A total of 108 (84.4%) patients received breast-conserving surgery, and 75 (58.6%) patients underwent axillary lymph node dissection. There were 122 (95.3%) patients with a clinical tumor diameter larger than 2.0 cm, and there were 87 (68.0%) patients with clinical lymph node metastases. The ER positivity rate was 35.2% (45 cases). Histological response grades were 0, 1a, 1b, 2a, 2b, and 3 in 0 (0.0%), 5 (3.9%), 12 (9.4%), 17 (13.3%), 11 (8.6%), and 83 (64.8%) patients, respectively. The pCR rate of the cohort was 64.8%. The RFS of those with pCR was significantly better than that of those with no pCR in the current study (X^2^ = 7.23, *p* = 0.0072). As shown in Table S2, pCR was significantly associated with ER negativity (*p* < 0.0001), PgR negativity (*p* = 0.00012), high (≥30%) Ki67 labeling index (*p* = 0.0090), and histological grade 3 (*p* = 0.0056).

### The relationship between TILs grade in pretreatment primary tumors and clinicopathological factors including pathological response to NAC with trastuzumab

**Figure 1:**
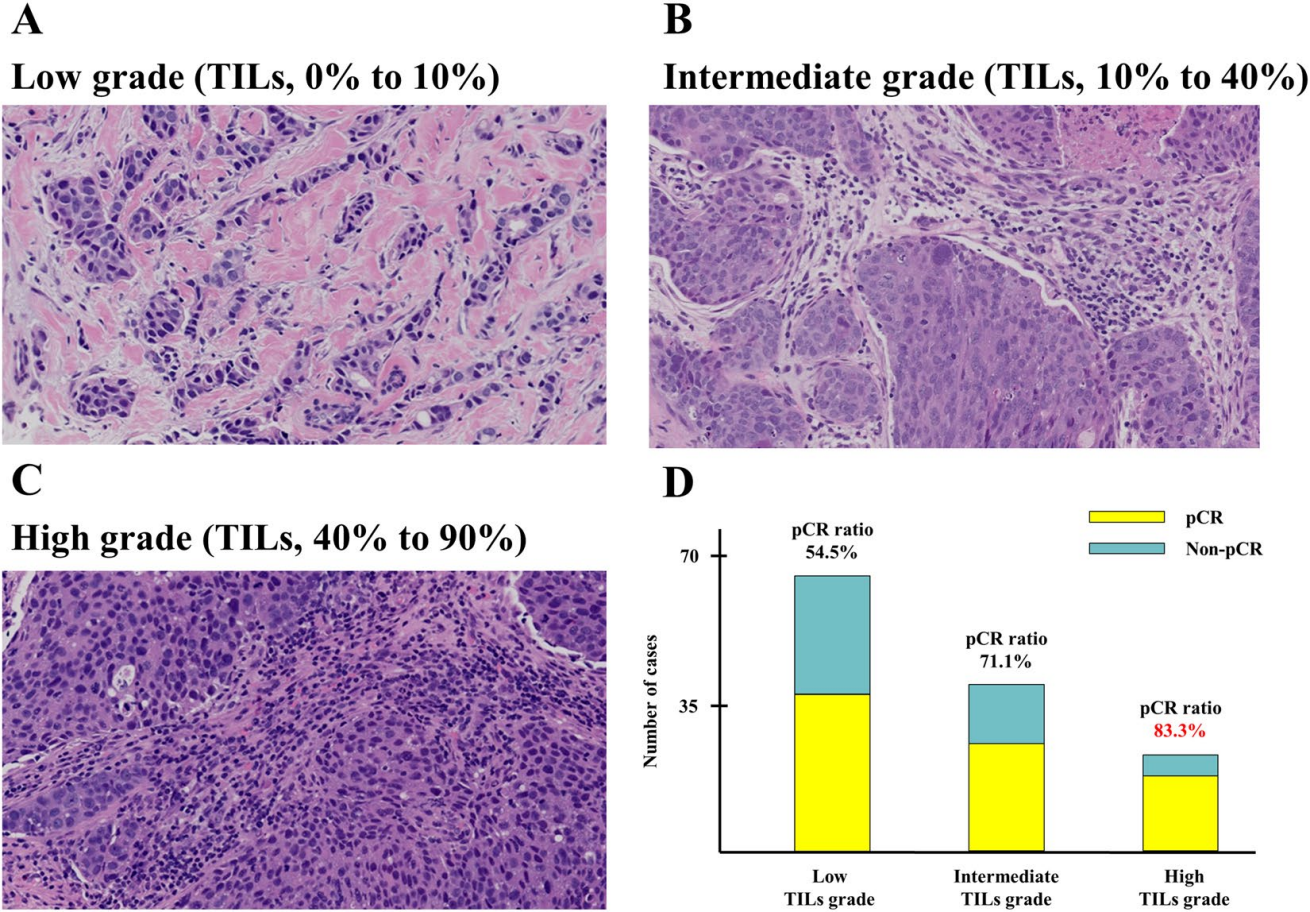
Representative images of tumor-infiltrating lymphocytes (TILs) in pretreatment tumor specimens in HER2-psitive breast cancer patients. (**A**) Low-grade TILs: a few lymphocytes in stromal tissue surrounding the cancer nests; (**B**) intermediate-grade TILs; (**C**) high-grade TILs: numerous lymphocytes are present in stromal tissue adjacent to the cancer nests. (**D**) Relationship between TILs grade and pathological complete response (pCR) to neoadjuvant chemotherapy with trastuzumab.

**Figure 2:**
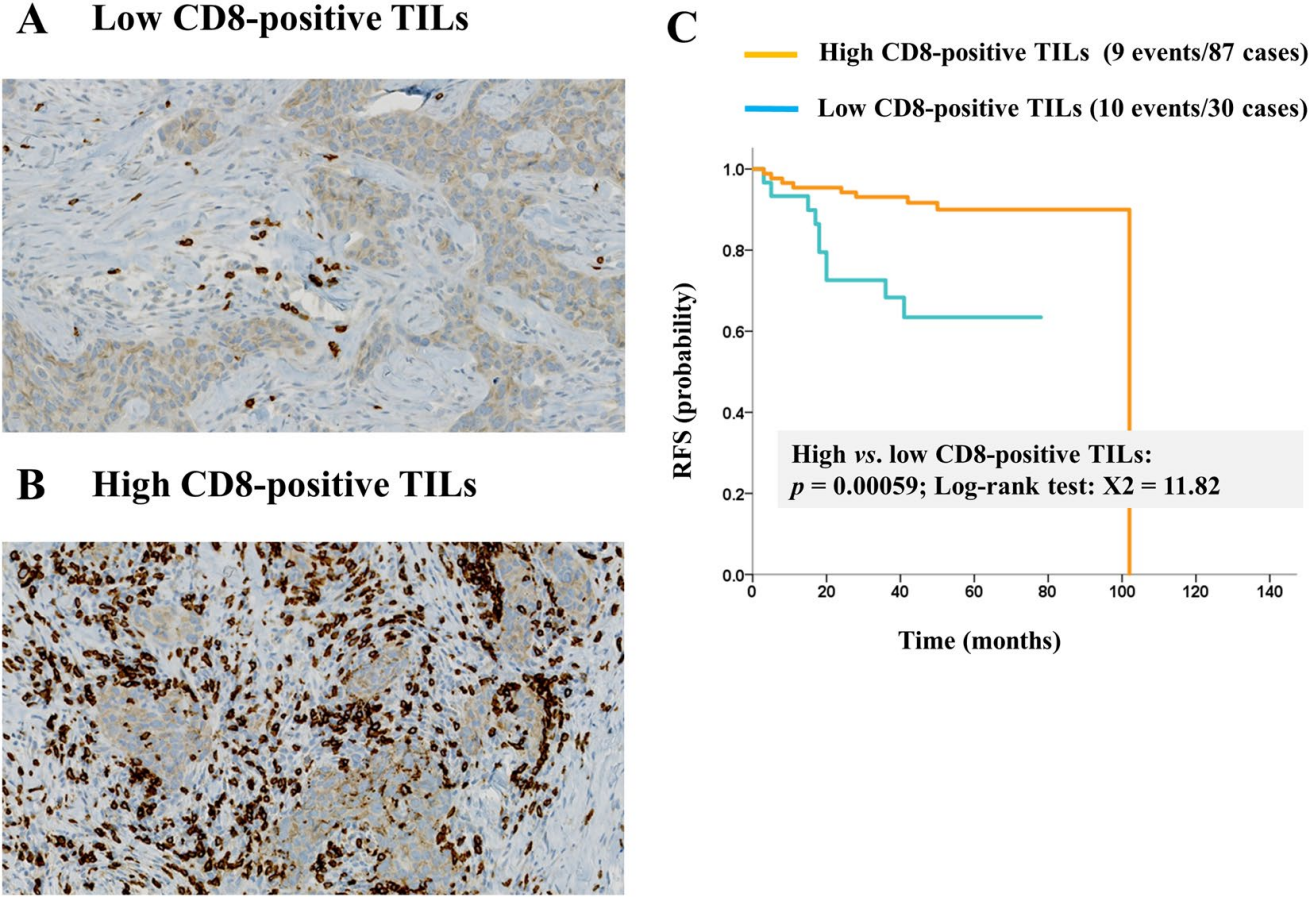
CD8 expression of tumor-infiltrating lymphocytes (TILs) in primary tumor and cumulative survival rates stratified by CD8-positive TILs density. (**A**) A case with low CD8-positive TILs, and (**B**) a case with high CD8-positive TILs. (**C**) Relapse free survival of the cases with low CD8-positive TILs was significantly worse than those with high CD8-positive TILs.

The entire cohort of 128 patients were categorized into those with low, intermediate, and high TILs grades based on the analysis of pretreatment tumor specimens (Fig. 1A–C). Briefly, 51.6%, 29.7%, and 18.8% of the primary tumors had low, intermediate, and high TILs grades, respectively (Table 1). As shown in Fig. 2A,B, the degrees of CD8-positive TILs were evaluated immunohistochemically. The number of patients with high CD8-postive TILs was 87 (74.4%) and those with low CD8-positive TILs was 30 (25.6%).

**Table 1:** Comparison of tumor-infiltrating lymphocytes grades in primary and residual tumors after neoadjuvant chemotherapy with trastuzumab.

TILs grade	Pretreatment tumors		Residual tumors	
	*N*	%	*N*	%
Low (TILs, 0% to 10%)	66	51.6	28	62.2
Intermediate (TILs, 10% to 40%)	38	29.7	8	17.8
High (TILs, 40% to 90%)	24	18.8	9	20.0
Total	128	100	45	100

Abbreviations: TILs: tumor-infiltrating lymphocytes.

The distribution of TILs grades in the pCR and non-pCR groups is presented in Table 2. The rate of pCR was correlated significantly with TILs grade (*p* = 0.026). The pCR rate in patients with high TILs grade was 83.3%, which was significantly higher than that in those with low TILs grade (54.5%; Fig. 1D), however, not significantly associated with CD8-positive TILs density (high, 70.1% vs. low, 56.7%; *p* = 0.18; Table 2).

**Table 2:** Comparison of tumor infiltrating lymphocytes (TILs) and CD8-positive TILs grades in primary tumors between the pCR and non-pCR groups.

Factors		pCR group	Non-pCR group	pCR ratio
		*N*	*N*	%
TILs	Low	36	30	54.5
	Intermediate	27	11	71.1
	High	20	4	83.3
CD8-positve TILs	Low	17	13	56.7
	High	61	26	70.1
Total		83	45	64.8

Abbreviations: TILs, tumor infiltrating lymphocytes; pCR, pathological complete response.

Among 24 patients with high TILs grade, 20 achieved pCR, whereas 2 patients had a histological grade 2a or 2b, and 2 patients had a histological grade 1a or 1b. Furthermore, high TILs grade was significantly associated with ER negativity (*p* = 0.015), PgR negativity (*p* = 0.037), high (≥30%) Ki67 labeling index (*p* = 0.031), and histological grade 3 (*p* = 0.033; Table S3).

### Changes in TILs grades between primary and residual tumors in patients with non-pCR

**Figure 3:**
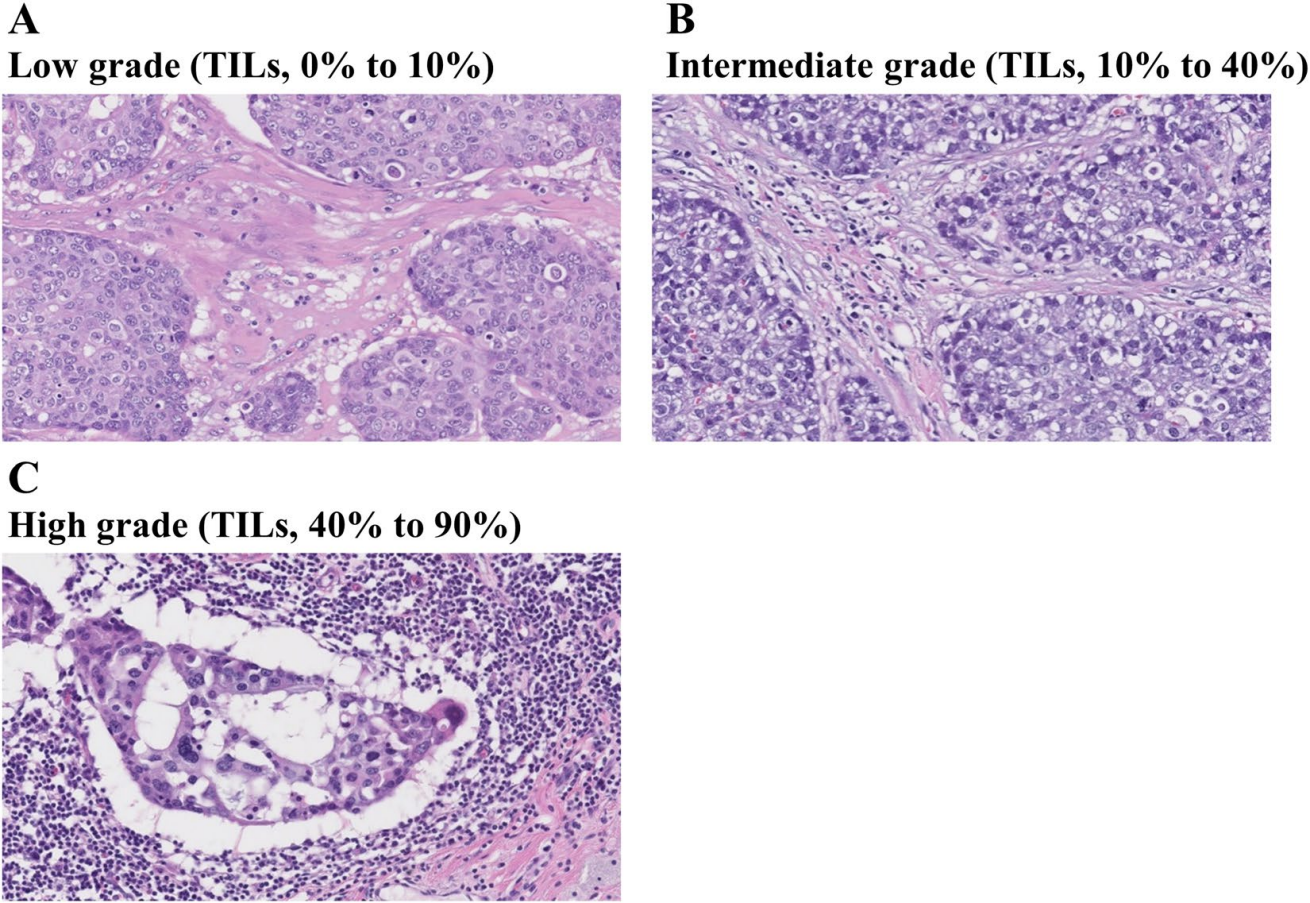
Representative images of tumor-infiltrating lymphocytes (TILs) in residual tumor specimens after neoadjuvant chemotherapy with trastuzumab. (**A**) Low-grade TILs; (**B**) intermediate-grade TILs; (**C**) high-grade TILs: numerous lymphocytes are distributed adjacent to the degenerated cancer cells.

The 45 patients in the non-pCR group were stratified into those with low, intermediate, and high TILs grades based on the analysis of the residual tumor specimens (Fig. 3). As shown in Table 1, 62.2%, 17.8%, and 20.0% of the patients who did not achieve pCR had residual tumors with low, intermediate, and high TILs grades, respectively. Changes in TILs grades between the primary and residual tumors in the non-pCR group are summarized in Table 3. The TILs grades were higher in the residual tumor compared with those in the primary tumor in 9 (20%) patients. Additionally, this group included 3 and 6 patients with histological grades 1a or 1b and 2a or 2b, respectively. Finally, there was no correlation between histological grade and increase in the TILs grade in the residual tumor.

**Table 3:** Changes in tumor-infiltrating lymphocytes grades between primary and residual tumors in the non-pCR group.

	*N*	%
Increasing TILs grade	9	20.0
Low to High	2	4.4
Intermediate to High	3	6.6
Low to Intermediate	4	8.8
Stable TILs grade	32	71.1
Stable with High	4	8.9
Stable with Intermediate	4	8.9
Stable with Low	24	53.3
Decreasing TILs grade	4	8.9
High to Intermediate	0	0
High to Low	0	0
Intermediate to Low	4	8.9
Total	45	100

Abbreviations: TILs, tumor-infiltrating lymphocytes: pCR, pathological complete response.

### Survival rates stratified by TILs grade in primary and residual tumors

**Figure 4:**
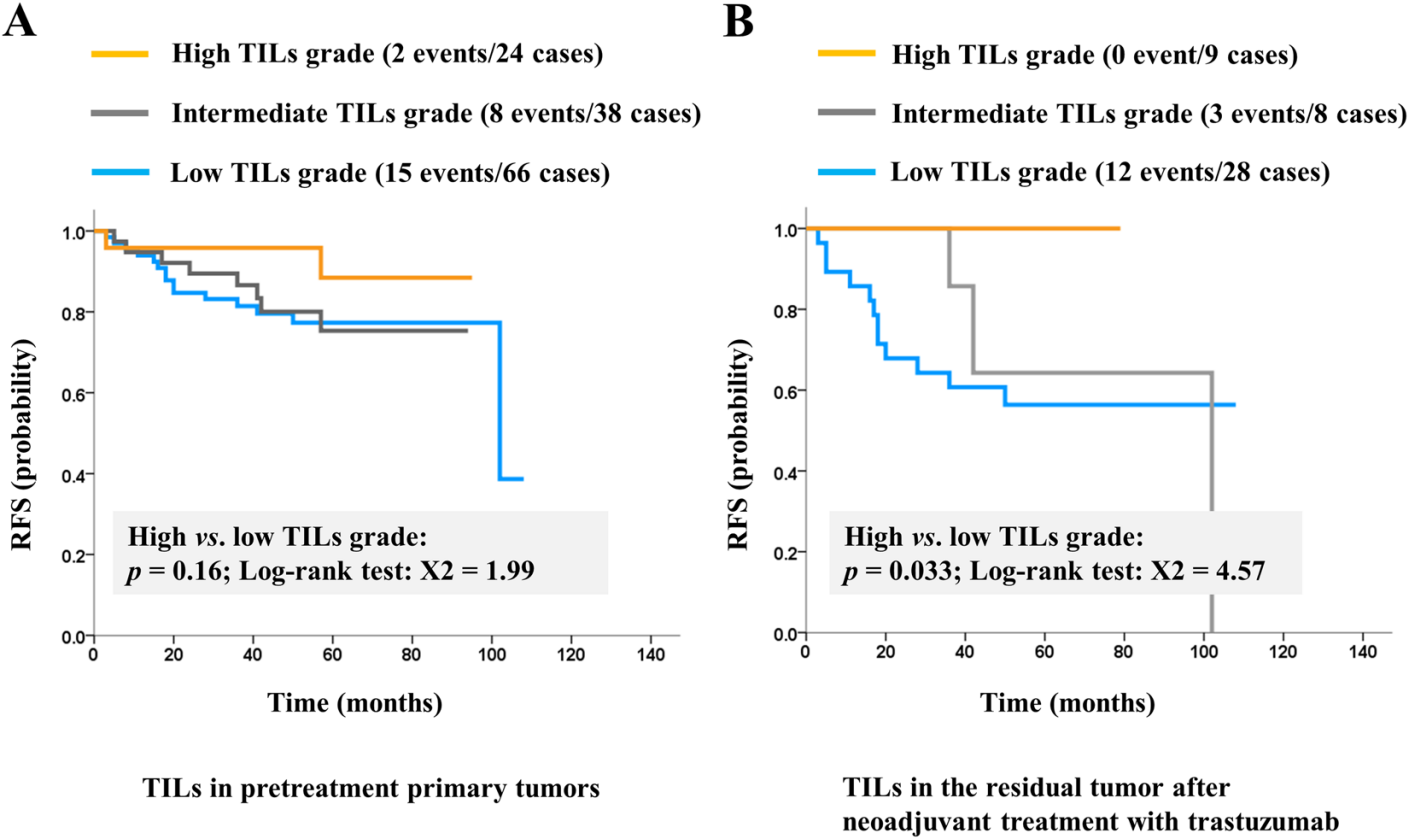
Survival curves stratified by tumor-infiltrating lymphocytes (TILs) grades. (**A**) Recurrence-free survival (RFS) is not significantly different between the high-TILs and the low-TILs group in pretreatment tumor specimens, whereas (**B**) the RFS of the high-TILs group was significantly better than that of the low-TILs group in residual tumors of patients without pCR.

In the entire cohort of 128 patients, the median Relapse-free survival (RFS) was 53 months (range, 3 to 108 months). In the 128 patients, TILs grade of the primary tumor was not a prognostic factor (X^2^ = 1.99, *p* = 0.16; Fig. 4A). However, the survival of high CD8-positive TILs group was significantly better than that of low CD8-positive TILs group (X^2^ = 11.82, *p* = 0.00059; Fig. 2C).

Among the 45 patients who did not achieve pCR, the RFS was significantly better in those with high TILs grade in the residual tumor than in those with low TILs grade in the residual tumor (X^2^ = 4.57, *p* = 0.033; Fig. 4B). There were no patients with recurrence during the follow-up period among those with a high TILs grade in the residual tumor.

## Discussion

The current study analyzing the role of TILs grade in HER2-positive breast cancer revealed that a high TILs grade in the primary tumor was associated with a significantly higher pCR rate. Furthermore, the TILs grade was increased after treatment compared with the pretreatment TILs status in 20.0% of the patients who did not achieve pCR after preoperative treatment. A high TILs grade in the residual tumor was also associated with a significantly better prognosis, compared with the residual tumors with a low TILs grade.

Denkert *et al*.^12^ reported that pathological response rate after preoperative drug treatment was associated with TILs grade in HER2-positive breast cancer. Specifically, they found that the pCR rate was significantly higher in the group with high TILs expression than that with low TILs expression in HER2-positive patient with NAC with trastuzumab and lapatinib simultaneously^12^.

In present study, all enrolled cases received trastuzumab for a total of 24 cycles with NAC consisting of paclitaxel or docetaxel followed by anthracycline. Buzdar *et al*.^13^ and Kurozumi *et al*.^4^ previously conducted this regimen of neoadjuvant treatment with trastuzumab on HER2-positive patients and achieved high pCR rate. However, the predictive factors of TILs in patients with HER2-positive breast cancer receiving this trastuzumab supplemented NAC regimens have not been clarified. Current findings suggested TILs expression as a potential predictor of the outcomes in preoperative NAC combined with 24 cycles of trastuzumab in HER2-positive breast cancer.

Several studies suggested that TILs expression in residual tumors following preoperative chemotherapy might be an important factor in evaluating the sensitivity of cancer to chemotherapeutic agents. In 2014, Dieci *et al*.^14^ compared the TILs expression levels before and after NAC among patients who did not achieve pCR and reported significantly better prognoses in the group with high TILs expression in residual tumors than in the group with low TILs expression. In addition, Miyashita *et al*.^15^ revealed in 2015 that a higher CD8/FOXP3 ratio in TILs following NAC was associated with better prognosis. Many studies reported that prognosis was worse in patients who did not achieve pCR following NAC than in those who achieved pCR^2,4,16,17^; however, it might be feasible to identify those patients who might achieve good prognosis among those who fail pCR by evaluating TILs expression in the residual tumor.

In 2014, the International Working Group created guidelines for the evaluation of TILs^18^ which stated that TILs could be evaluated in needle biopsy specimens. However, because of the heterogeneity of TILs within the tissue, evaluation must be accomplished after ascertaining the distribution and level of TILs expression across all needle biopsy specimens. Moreover, although several reports^14,15^ are considered useful for their evaluations of TILs in residual tumors following preoperative chemotherapy, a strong pathological therapeutic effect can also leave behind a diffuse scattering of residual cancer cells. Because of the extreme difficulty in determining the suitable area for evaluation, this approach to evaluate TILs expression is not included in the guidelines. Therefore, evaluation of TILs in residual tumors following NAC requires the establishment of an optimal evaluation method by additional studies.

Trastuzumab, a molecular-targeted drug, binds to the surface HER2 antigen to inhibit its dimerization, thereby inhibiting the transduction of signals to the phosphatidylinositol 3-kinase cascade downstream of HER2^19,20^. Trastuzumab also triggers an antitumor effect by antibody-dependent cellular cytotoxicity (ADCC) to stimulate natural killer cells^21,22^. In the present study, TILs expression within breast cancer tissues was enhanced after NAC with trastuzumab compared with the pretreatment evaluation in 20% of the non-pCR patients, suggesting that trastuzumab might stimulate the lymphocytes within the tumor by a mechanism that is distinct from ADCC.

Trastuzumab-TDB, similar to trastuzumab, has two arms, one binding to HER2 on breast cancer cells and the other binding to CD3 on T cells, thereby triggering an anti-tumor effect by inducing a strong intratumor immune response. Recent efforts have been focused on bispecific antibodies such as trastuzumab-TDB that can utilize immune responses^23^. Perez *et al*.^24^ used a large transcriptomic dataset to reveal that gene subsets associated with immune function were strongly correlated with the beneficial effect of trastuzumab in HER2-positive breast cancer. These studies suggest that trastuzumab is strongly associated with the activation of killer T cells as well as ADCC. In present study, TILs grade of the primary tumor was not a significant prognostic factor. However, the prognosis of the cases with high CD8-positive TILs density was significantly better than those with low CD8-positive TILs density. For effective use of molecular-targeted drugs and immune checkpoint inhibitors involved in intratumor immune responses in the future, more in-depth analyses of differences in the characteristics of immune cells that make up TILs that are present before and after NAC with trastuzumab using biomarkers, such as programmed death 1, programmed death-ligand 1, CD4, CD8, and FOXP3, are needed to evaluate the utility of TILs expression in assessing outcomes in breast cancer patients.

In conclusions, TILs status of primary tumors might predict pCR to NAC in combination with trastuzumab in HER2-positive breast cancer. Patients with high TILs grades in residual tumors exhibited better prognosis than those with low TILs grades in residual tumors. Evaluation of TILs in the residual tumor after NAC with trastuzumab might be necessary to predict patients who might achieve good prognosis among those without pCR.

## Methods

### Patient background

Among all patients who underwent surgery at the Division of Breast Surgery in Saitama Cancer Center between 2005 and 2011, 128 consecutive patients with HER2-positive invasive breast cancer who received NAC with trastuzumab were included in this study.

Immunohistochemistry and *in situ* hybridization were performed as described previously^4,25^. Briefly, the following antibodies were used in immunohistochemical staining for subtype determination: estrogen receptor (ER; 1D5; DAKO, Copenhagen, Denmark), progesterone receptor (PgR; PgR636; DAKO), and HER2 (HercepTest; DAKO). HER2 amplification was achieved using an automated slide processing system (BenchMark^®^ XT; Ventana Medical Systems, Tucson, Arizona, USA) with dual *in situ* hybridization (DISH; INFORM HER2 Dual ISH DNA Probe Cocktail Assay; Roche, Basel, Switzerland). Expression levels of ER, PgR, and HER2 were determined in accordance with the American Society of Clinical Oncology/College of American Pathologists criteria. Specimens with a nuclear staining rate of at least 1% were considered positive for ER and PgR. Evaluation of HER2 immunohistochemical staining was based on four grades corresponding to scores of 0, 1+, 2+, and 3+, which depended on staining intensity of cell membranes. Only specimens with a score of 2+ by HER2 immunohistochemical staining were evaluated for gene amplification by DISH, and those with a HER2 immunohistochemical score of 3+ or 2+ and positive for HER2 amplification by DISH were defined as HER2-positive breast cancer^4^.

The details of NAC were as follows: 12 cycles of paclitaxel (80 mg/m^2^) every week or 4 cycles of docetaxel (75 mg/m^2^) every 3 weeks, followed by 4 cycles of FEC 75 (500 mg/m^2^ 5-fluorouracil, 75 mg/m^2^ epirubicin, and 500 mg/m^2^ cyclophosphamide) every 3 weeks. All patients also received 4 mg/kg trastuzumab on day 1 of the treatment and 2 mg/kg trastuzumab every week thereafter, for a total of 24 cycles. Trastuzumab was used for 6 months as adjuvant therapy. In addition, ER-positive breast cancer patients underwent postoperative endocrine therapy with tamoxifen or an aromatase inhibitor. We reported the utility as well as the eligibility and exclusion criteria for this protocol previously^4^.

All patients provided informed consent to participate in the study, which was approved by the Institutional Review Board of Saitama Cancer Center (Reference number: 534) and conducted in full compliance with the Declaration of Helsinki.

### Evaluation of tumor-infiltrating lymphocytes

Hematoxylin/eosin-stained samples were prepared from formalin-fixed, paraffin-embedded sections of 4 μm slices from core needle biopsy specimens in all patients as well as surgical specimens in non-pCR patients. A pathologist specialized in breast pathology used an optical microscope at 200–400 × magnification to determine whether mononuclear immune cells interposing between tumor nests were stromal TILs. Other immune cells present in tumor specimens were not evaluated. Considering the heterogeneity of TILs within tissue, the distribution of TILs was evaluated using all core needle biopsy samples. In surgical specimens from non-pCR patients, residual TILs in sites with the highest residual tumor concentration were evaluated. If the pathological effect of treatment was strong but the amount of residual tumor was low, lymphocytes aggregation surrounding degenerating cancer cells were evaluated as TILs. The TILs grade, as previously reported^26^, was categorized into three groups by modifying the International Working Group criteria^18^: low (TILs: 0% to 10%), moderate (TILs: 10% to 40%), and high (TILs: 40% to 90%).

The immunohistochemical expression of CD8 in TILs was evaluated in primary tumors using core needle biopsy specimens. The source of the primary antibody of CD8 was FLEX Monoclonal Mouse Anti-Human CD8, Dako, Copenhagen, Denmark. Staining was performed automatically using an automated immunohistochemistry instrument (BenchMark® XT, Ventana Medical Systems, Inc., Tucson, Arizona). High CD8 expression was defined as number of CD8-positive TILs > 25 in one high power field.

### Evaluation of histological response

Grading of the pathological response to NAC was performed in accordance with the Japanese Breast Cancer Society criteria^4^, which categorizes pathological response into six histological grades (0, 1a, 1b, 2a, 2b, and 3) based on the degree of morphological changes in the primary tumor as a result of NAC treatment. Grade 1a was defined as “mild change in cancer cells regardless of the area or marked changes in cancer cells in less than one-third of total cancer cells,” whereas grade 1b was defined as “marked changes in one-third or more but less than two-thirds of cancer cells.” Grade 2a was defined as “marked changes in two-thirds or more of cancer cells, but with clear cancer nests,” and grade 2b was defined as “an effect extremely close to a complete response (grade 3), but with a very small amount of residual cancer cells.” Finally, grade 3 was defined as “disappearance of all cancer cells” and was equivalent to pCR in the NSABP B-18 trial^1^. Residual noninvasive cancers or axillary lymph node metastases are not considered in the determination of the Japanese Breast Cancer Society grades.

### Statistical analysis

Associations of TILs grades with pCR rate and clinicopathological factors were evaluated using the chi squared and Fisher’s exact tests. In addition, the association between the TILs grade and prognosis based on RFS was determined using the Kaplan Meier method and the log rank test. RFS was defined as the period from the day of surgery to the day of relapse in any organ including ipsilateral breast recurrence. Statistical analyses were conducted using the SPSS statistical software version 22.0 (IBM, Armonk, New York, USA), and a *p* value of 0.05 or less was considered to indicate a significant difference.

### Ethical approval and informed consent

This study was approved by the Institutional Review Board of Saitama Cancer Center (Reference number: 534). All procedures performed in studies involving human participants were in accordance with the ethical standards of the institutional and/or national research committee and with the 1964 Helsinki declaration and its later amendments or comparable ethical standards. Informed consent was obtained from all individual participants included in the study.

## Supplementary information

Supplementary Information

## Data Availability

The datasets generated and/or analyzed during the current study are not publicly available due to the regulation of the Institutional Review Board of the Saitama Cancer Center but are available from the corresponding author on reasonable request.
