# Supplementary material for: Prognostic utility of tumor-infiltrating lymphocytes in residual tumor after neoadjuvant chemotherapy with trastuzumab for HER2-positive breast cancer
Source: Sci Rep. 2019 Feb 7;9:1583. doi: 10.1038/s41598-018-38272-1 (PMC6367461; doi:10.1038/s41598-018-38272-1)
Supplement: Supplementary file 1 — Supplementary Information [file 41598_2018_38272_MOESM1_ESM.pdf]

Supplementary Information

Prognostic utility of tumor-infiltrating lymphocytes in residual tumor after neoadjuvant chemotherapy with trastuzumab for HER2-positive breast cancer

Sasagu Kurozumi, Kenichi Inoue, Hiroshi Matsumoto, Takaaki Fujii, Jun Horiguchi, Tetsunari Oyama, Masafumi Kurosumi, and Ken Shirabe

Table S1. Patients and tumor characteristics

Age range (years)		Clinical tumor size	
<40	20	T1	6
40≤ and <60	82	T2	80
≥60	26	T3	26
Menopausal status		T4	16
Premenopausal	51	Clinical nodal status	
Postmenopausal	77	N0	41
Type of breast surgery		N1	58
Breast-conserving surgery	108	N2	17
Mastectomy	20	N3	12
Axillary surgery		Clinical TNM stage	
Sentinel lymph node biopsy alone	53	I	2
Axillary lymph node dissection	75	IIA	37
ER status		IIB	37
ER-positive	45	IIIA	29
ER-negative	83	IIIB	11
		IIIC	12
Abbreviations: ER, estrogen receptor			

Table S2. Relationship between pathological complete response and clinicopathological factors

		pCR	Non-pCR	pCR ratio (%)	*p*
		N	*N*		
ER	Negative	65	18	78.3	<0.0001
	Positive	18	27	40.0	
PgR	Negative	69	23	75.0	0.00012
	Positive	14	22	38.9	
Ki-67 labeling index	High (≥30%)	66	26	71.7	0.0090
	Low (<30%)	17	19	47.2	
Histological grade	Grade 3	76	33	69.7	0.0056
	Grade 1/2	7	12	36.8	
Clinical tumor size	T 3/4	27	19	58.7	0.28
	T 1/2	56	26	68.3	
Clinical nodal status	Positive	56	31	64.4	0.87
	Negative	27	14	65.9	

Abbreviations: ER, estrogen receptor; PgR, progesterone receptor; pCR, pathological complete response

Table S3. Correlations of tumor-infiltrating lymphocytes grades in the primary tumor and clinicopathological factors

		Low (TILs: 0%–10%)	Intermediate (TILs: 10%–40%)	High (TILs: 40%–90%)	*p*
ER	Negative	31	29	19	0.015
	Positive	35	9	5	
PgR	Negative	41	32	19	0.037
	Positive	25	6	5	
Ki-67 labeling index	High (≥30%)	41	30	21	0.031
	Low (<30%)	25	8	3	
Histological grade	Grade 3	51	36	22	0.033
	Grade 1/2	15	2	2	
Clinical tumor size	T 3/4	21	15	10	0.60
	T 1/2	45	23	14	
Clinical nodal status	Positive	39	28	20	0.062
	Negative	27	10	4	

Abbreviations: TILs, tumor-infiltrating lymphocytes; ER, estrogen receptor; PgR, progesterone receptor
